# New bacteriophage-derived lysins, LysJ and LysF, with the potential to control *Bacillus anthracis*

**DOI:** 10.1007/s00253-023-12839-z

**Published:** 2024-01-09

**Authors:** Aleksandra Nakonieczna, Agnieszka Topolska-Woś, Małgorzata Łobocka

**Affiliations:** 1 Military Institute of Hygiene and Epidemiology, Biological Threats Identification and Countermeasure Center, 24-100 Puławy, Poland; 2 SDS Optic, EcoTech Complex, 20-612 Lublin, Poland; 3 Institute of Biochemistry and Biophysics of the Polish Academy of Sciences, 02-106 Warsaw, Poland

**Keywords:** Bacteriophage, Endolysin, *Bacillus anthracis*, Lytic activity, Optical density, Spot test

## Abstract

**Abstract:**

*Bacillus anthracis* is an etiological agent of anthrax, a severe zoonotic disease that can be transmitted to people and cause high mortalities. Bacteriophages and their lytic enzymes, endolysins, have potential therapeutic value in treating infections caused by this bacterium as alternatives or complements to antibiotic therapy. They can also be used to identify and detect *B. anthracis.* Endolysins of two *B. anthracis Wbetavirus* phages, J5a and F16Ba which were described by us recently, differ significantly from the best-known *B. anthracis* phage endolysin PlyG from *Wbetavirus* genus bacteriophage Gamma and a few other *Wbetavirus* genus phages. They are larger than PlyG (351 vs. 233 amino acid residues), contain a signal peptide at their N-termini, and, by prediction, have a different fold of cell binding domain suggesting different structural basis of cell epitope recognition. We purified in a soluble form the modified versions of these endolysins, designated by us LysJ and LysF, respectively, and depleted of signal peptides. Both modified endolysins could lyse the *B. anthracis* cell wall in zymogram assays. Their activity against the living cells of *B. anthracis* and other species of *Bacillus* genus was tested by spotting on the layers of bacteria in soft agar and by assessing the reduction of optical density of bacterial suspensions. Both methods proved the effectiveness of LysJ and LysF in killing the anthrax bacilli, although the results obtained by each method differed. Additionally, the lytic efficiency of both proteins was different, which apparently correlates with differences in their amino acid sequence.

**Key points:**

*• LysJ and LysF are B. anthracis-targeting lysins differing from lysins studied so far*

*• LysJ and LysF could be overproduced in E. coli in soluble and active forms*

*• LysJ and LysF are active in killing cells of B. anthracis virulent strains*

**Supplementary Information:**

The online version contains supplementary material available at 10.1007/s00253-023-12839-z.

## Introduction

Infections caused by multidrug-resistant bacteria (MDR) have become a significant clinical problem in recent years. The perspective of returning to a pre-antibiotic era bears a risk of not only increased death rates as a major concern but also of increased healthcare costs (Durai et al. 2010). *Bacillus anthracis* is an etiological agent of anthrax—a severe zoonotic disease that can be transmitted to people, especially those exposed occupationally. The pathogen can cause 86–89% mortality in untreated people when inhaled and 25–60% in a gastrointestinal form (Ghosh and Goel 2012) and is considered one of the greatest biowarfare threats. Due to its ability to form endospores, it is especially hard to fight. Anthrax starts rapidly with a high temperature, chills, and a strong cough with hemoptysis. In addition, patients have acute shortness of breath and cyanosis. The clinical picture of anthrax resembles severe pneumonia. The treatment procedure currently recommended by the CDC relies on broad-spectrum antibiotics, including prevention of inhalational *B. anthracis* infection (penicillin, ciprofloxacin, and doxycycline) (Brook 2002) or antitoxin therapy. However, to prevent delayed spore germination, administration of an antibiotic may take up to 60 days and may require intravenous administration. Unfortunately, burying the animals treated with antibiotics in the ground promotes the development of antibiotic resistance in bacteria, which may decrease the effectiveness of treatment. Additionally, it can be assumed that strains of *B. anthracis* with naturally or artificially developed antibiotic resistance may potentially be used in a bioterrorist attack as a weapon of mass destruction (Park et al. 2018). Therefore, it is in the public interest to develop new rapid antimicrobial agents against this pathogen with different mechanisms of action than commonly used antibiotics. Among them, bacteriophages and their derivatives—endolysins and antimicrobial peptides—are of great interest (Mirski et al. 2019). Phages are viruses that specifically infect bacteria and play a key role in ecology, the formation of microbial diversity in nature, and the evolution of bacteria (Mäntynen et al. 2021). Endolysins are phage-encoded hydrolases produced as late proteins in phage-infected bacterial cells at the end of a lytic cycle. They are directly responsible for disrupting bacterial cells and releasing progeny viral particles by cutting the bonds crucial for maintaining a peptidoglycan structure (Wang et al. 2000; Fischetti 2008). Lysins have certain advantages over antibiotics. The lack of resistance development to lysins seems to be the most important one (Gondil et al. 2020; Abdelrahman et al. 2021; Arroyo-Moreno et al. 2022). Moreover, the lysins can lyse cells of antibiotic-sensitive as well as antibiotic-resistant bacteria and are not inhibited by antibodies (Horgan et al. 2008). Many lysins have already been used to treat bacterial infections in humans and animals (Abdelkader et al. 2019; Gondil et al. 2020). Lysin therapy can also be combined with antibiotic therapy, and in certain cases, such mixed therapies are recommended (Wittekind and Schuch 2016).

Here, we describe lysins derived from phages specific to *Bacillus anthracis* that were identified by us recently (Nakonieczna et al. 2022). We show that the new lysins differ from those identified previously in their sequence and predicted structure and that they are active in killing *Bacillus* anthracis cells of vaccine as well as various virulent strains.

## Materials and methods

### Bacterial strains and growth conditions

Bacterial strain *E. coli* NEB 5-alfa (New England Biolabs, NEB, Ipswich, MA, USA) was used for cloning experiments and plasmid extraction, and *E. coli* BL21 (DE3) (NZYtech, Lisbon, Portugal) was used for the production of recombinant proteins. They were grown in LB (Luria–Bertani broth) and on LA (Luria–Bertani agar) media. The *Bacillus* genus strains that were used in this study for endolysins activity assays are listed in Table 2. They were grown in TSB (Trypticase soy broth) and on TSA (Trypticase soy agar) or Columbia agar media when indicated. One of the *B. anthracis* strains used was the attenuated vaccine strain (*B. anthracis* Sterne 34F2), and five others were fully virulent strains cultured in a biosafety level 3 laboratory. *Bacillus* sp*. Ba 813* + strains are transition strains belonging to the *Bacillus cereus* group. They do not carry plasmids but have a chromosomal anthrax marker gene *Ba 813* in their DNA (Niemcewicz and Bartoszcze 2006a). Their origin is not confirmed, but they might have originated from *B. anthracis* (Niemcewicz and Bartoszcze 2006b).

### Bioinformatic analysis

Genes encoding endolysins of J5a and F16Ba bacteriophages were identified in the genomes of these phages (GenBank accession numbers: MT745955 and MT745954, respectively), as reported previously (Nakonieczna et al. 2022). Basic Local Alignment Search Tool (BLAST) was used to determine the similarities of their products with PlyG and other lysins of *B. anthracis* phages. Expasy was used to compute the molecular weight of both endolysins and their truncated derivatives (https://web.expasy.org/compute_pi/), LysJ and LysF, respectively. Signal peptides were predicted using SignalP 5.0 (https://services.healthtech.dtu.dk/service.php?SignalP-5.0) (Bendtsen et al. 2004) and other online tools: PrediSi (http://www.predisi.de/), TOPCONS (https://topcons.cbr.su.se/), Phobius (https://phobius.sbc.su.se/), and SOSUIsignal (https://harrier.nagahama-i-bio.ac.jp/sosui/sosuisignal/sosuisignal_submit.html). Pfam (InterPro) server (https://www.ebi.ac.uk/interpro/) and HHpred (https://toolkit.tuebingen.mpg.de/tools/hhpred) were used to predict the secondary structure and domain structure of both endolysins.

### Lysins homology modeling and structure assessment

Models of the lysins were computed by the SWISS-MODEL server homology modeling pipeline (Waterhouse et al. 2018) which relies on ProMod3 (Studer et al. 2020), an in-house comparative modeling engine based on OpenStructure (Biasini et al. 2013). In addition to the PDB-based SMTL (SWISS-MODEL Template Library), SWISS-MODEL also searches the AlphaFold DB (Varadi et al. 2022) for templates with high sequence identity (≥ 70%). For each of the analyzed lysins, all models were analyzed for the highest coverage, highest GMQE (Global Model Quality Estimate) score, and structure assessment with Molprobity score and, among others, Ramachandran plot.

For PlyG (1–233 aa), three models have been generated, covering amino acid sequence residues: 1–233, 1–165, and 151–233, respectively. The model with the largest coverage was based on the AlphaFold DB model of A0A1J9VD13_BACAN (gene: A0A1J9VD13_BACAN, organism: *Bacillus anthracis*) and its performance and probability were the best among other models with GMQE of 0.94 and MolProbity Score 1.36, with 95.24% of residues falling into Ramachandran Favored. For LysF (1–325), a total of 6 models have been generated with a large coverage of amino acid residues 2–325, N-terminal domain with residues 5–168, and 4 models within the C-terminal domain of the protein: 189–318, 190–317, 185–295, and 193–293, respectively. The best-performing model used the AlphaFold DB model of A0A6I2A7M9 (gene: unknown, organism: unknown) and covered almost the entire amino acid sequence. Its GMQE score was estimated at 0.87 with 96.06% sequence identity to a UniProtKB template and MolProbity Score 0.92 with 95.03% of residues falling into Ramachandran Favored. Finally, for LysJ (1–325), similarly to LysF, 6 models have been generated, with almost full coverage: 2–325, N-terminal domain: 6–168, and 4 models within the C-terminal domain: 189–318, 190–317, 191–317, and 193–293. GMQE score for the model with the largest coverage was estimated at 0.87 with 99.38% sequence identity to the Uniprot template: AlphaFold DB model of A0A6I2A7M9 (gene: unknown, organism: unknown). MolProbity Score was 0.94, with 94.72% of residues falling into Ramachandran Favored. For each lysin, the best-performing model has been selected and used for further comparison.

### Cloning experiments

Gibson Assembly (GA) method and NEBuilder HiFi DNA Assembly Cloning Kit (NEB) were used to clone into the expression vector pET30c( +) (Novagen) the J5a and F16Ba DNA fragments encoding the truncated versions of endolysins of these phages depleted of the signal peptide. In the vector, the cloned genes are enriched with the His-tag encoding sequences at their 3′-termini. Additionally, to avoid the interference of His-tag with the folding of cloned gene products, a linker composed of three glycine codons was added to each reverse primer designed for the amplification of inserts. The inserts and the vector were first PCR-amplified with Q5® High-Fidelity DNA Polymerase (NEB), and the obtained vector product was digested with DpnI restriction enzyme for 30 min at 37 °C to remove traces of the original template. Sequences of the used primers are listed in Table 1. Mixtures of the linear vector amplicon and respective insert amplicons were incubated for 15 min at 50 °C. Ligated constructs were then transformed into chemically competent *E. coli* NEB 5-alfa. The sequence correctness of plasmids isolated from transformants grown on a selective medium was verified by sequencing. Single plasmids of the correct sequence were transformed into *E. coli* expression strain BL21 (DE3).

**Table 1:** Primers used for the amplification of the vector and the inserts (genes encoding the modified endolysins). Bolded fragments in capitals indicate regions complementary to the vector sequence, and underlined fragments indicate regions complementary to the insert sequence. The sequence of the introduced linker is in italics. F and R indicate the orientation of the primers

Primers for insert (LysJ)	**GAAGGAGATATACATATG**gacagaatattaatcattcccgat
	**TCAGTGGTGGTGGTGGTGGTG***tcctccccc*cttcacatacacataggctt
Primers for insert (LysF)	**GAAGGAGATATACATATG**gacagagtattgatcattcccgat
	**TCAGTGGTGGTGGTGGTGGTG***tcctccccc*cttcacatacacataggctt
Primers for vector	**CATATGTATATCTCCTTCTTAAAGTTAAACAAAA**
	*gga***CACCACCACCACCACCACTGA**

### Endolysins expression and purification

LB with kanamycin (40 µg/ml) was inoculated with *E. coli* BL21 (DE3) transformants containing the recombinant vectors (with LysJ and LysF lysin genes) and incubated overnight at 37 °C with shaking. The overnight cultures were diluted 1:100 in fresh LB with kanamycin and incubated until the optical density (OD_600_) reached ~ 0.5. Next, isopropyl-β-D-1-thiogalactopyranoside (IPTG) was added to the cultures to the final concentration of 1 mM to induce the expression of recombinant genes. The cultures were incubated at 30 °C until the OD_600_ reached ~ 1.7, and centrifuged for 30 min at 5000 × g. The pellets were drained, weighed, and suspended in BugBuster lysis reagent (5 ml/1 g, Merck, Darmstadt, Germany) supplemented with Benzonase Nuclease (25 U/1 ml BugBuster, Merck), serine protease inhibitor PMSF (5 µl of conc. 200 mM/1 ml BugBuster) and lysozyme (1 KU/1 ml BugBuster). The cell suspensions were incubated on a shaking platform or rotating mixer at a low setting for 20 min at RT and centrifuged (20 min, 4 °C at 16 000 × g). The supernatants containing soluble proteins were filtered through a 0.45 µm Merck Millipore (Burlington, MA, USA) syringe filter with a PVDF membrane. The recombinant proteins were then purified on HisPur Ni–NTA Resin (Thermo Fisher Scientific, Waltham, MA, USA) in Qiagen columns according to the manufacturer’s instructions. The purification process was performed using imidazole elution (250 mM). The purity of the samples was assessed by SDS-PAGE, and the presence of histidine tag-containing proteins was verified by Western blotting with the use of the anti-His monoclonal antibody (penta·His HRP Conjugate, Qiagen, Hilden, Germany), according to the standard protocol. The antibodies conjugate was diluted 1000 × in TBST buffer supplemented with 5% skimmed milk. Elution fractions of a suitable purity were pooled and dialyzed overnight at 7 °C against 2 l of a dialysis buffer (50 mM Tris–HCl, 200 mM NaCl, 5% glycerol, pH 8.0) to remove residual imidazole. Samples were then concentrated using Amicon® Ultra Centrifugal filters (Merck) with a 30 kDa molecular weight cutoff. Protein concentrations were measured by the Bradford method (Bradford Reagent, ready-to-use, Thermo Fisher Scientific). The obtained proteins were aliquoted into PCR tubes and stored frozen at − 75 °C.

### Zymography

The enzymatic activity of purified LysJ and LysF in the lysis of *B. anthracis* cell walls was verified by zymography. First, the lysins at a concentration of 50 µg/ml each were mixed with 2 × concentrated Laemmli buffer, and the samples were heated for 5 min at 95 °C. Then, the samples were loaded on a 12% SDS PAGE gel supplemented with *B. anthracis* 34F2 cell wall preparation as the substrate for the enzymes. After the electrophoretic separation, the gel was washed in water for 30 min and then placed in renaturation buffer (50 mM Tris–HCl, 1% Triton X-100, pH 7.4) for a few hours or overnight until the appearance of cleared bands in the turbid background.

### Preparation of *B. anthracis* cell walls for zymography

To prepare the substrate for the endolysins in the zymogram, the overnight bacterial culture was diluted 100-fold in 250 ml of TSB medium and grown at 37 °C at 200 rpm until an OD_600_ of 1 was obtained. The culture was centrifuged at room temperature for 15 min at 10,000 × g. The pellet was washed with 250 ml of ultrapure water (Simplicity® UV Water Purification System, Merck Millipore), centrifuged under the same conditions, and resuspended in 30 ml of water. The cell suspension was autoclaved for 15 min at 121 °C and centrifuged again. The pellet was stored overnight at − 20 °C and, the following day, suspended in 3 ml of water and aliquoted into three previously weighed Eppendorf tubes. The opened tubes were placed in a Concentrator 5301 (Eppendorf, Hamburg, Germany) and dried at 30 °C for about 2 h. The dried pellets were weighed and suspended together in 1 ml of water.

### Plate lysis (spot) assay

The antibacterial activity of LysJ and LysF against the tested strains was initially assessed using the plate lysis assay. Briefly, the lysins were diluted in PBS to the following final concentrations: 0.05, 0.1, 0.25, 0.5, 0.75, 1.0, 1.5, and 2.0 mg/ml for LysJ and 0.1, 0.25, and 0.4 mg/ml for LysF. Spots (10 µl) were dropped on double-layered Petri dishes. The bottom layer consisted of TSA medium, and the upper layer contained *B. anthracis* cells in 0.7% soft agar. The plates were incubated face up for 4–6 h at 37 °C for the clear zone observations. The same protocol but with only one concentration of both lysins, 0.1 mg/ml, was performed for the remaining *Bacillus* strains. PBS without any lysin was used as a negative control.

### Turbidity reduction assay

The antibacterial activity of LysJ and LysF was measured also in liquid via the turbidity reduction assay. The experiments were performed in 96-well titration plates. Briefly, overnight bacterial cultures were diluted 100-fold in fresh medium and grown at 37 °C at 110 rpm until an OD_600_ of 0.4–0.5 (as measured in a reader used for this assay, Ultramark Microplate Imaging System reader, Bio-Rad, Hercules, CA, USA) in the working volume. The bacterial cells were then harvested by centrifugation for 8 min at 18 600 × g and resuspended in the same volume of 20 mM Tris–HCl, pH 8.0. An aliquot of 180 µl of each bacterial suspension was added to the well containing 20 µl of each lysin at a predetermined concentration. The turbidity measurements were taken at room temperature (RT) every 5 min for 45 min in total with the use of the reader. The measurements were performed in duplicate or triplicate. PBS without any lysin was used as a negative control. *B. anthracis* 34F2 strain was used for the first assessment of the lysins’ optical density reduction ability, and a broader range of lysins concentrations was used.

## Results

### Purification of modified versions of J5a and F16Ba phage endolysins

**Fig. 1:**
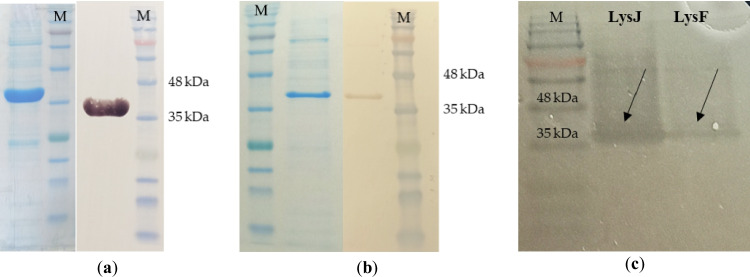
Identification of LysJ-(His × 6) (**a**) and LysF-(His × 6) (**b**), separated electrophoretically by SDS-PAGE, and detection of their activity (**c**). Left panels in (**a**) and (**b**) show the images of the respective proteins in SDS-PAGE. Right panels in (**a**) and (**b**) show Western blots of SDS-PAGE-separated respective proteins with anti-His-tag antibodies. Cleared bands in the zymogram shown in (**c**) indicate the enzymatic activity of LysJ and LysF against the cell wall of *B. anthracis* 34F2 strain (**c**). Perfect Tricolor (EurX) was used as a protein ladder (M). The total amounts of protein loaded in each well were the following: 15 µg (**a**), 5 µg (**b**), 0.5 µg (**c**)

Endolysins encoded by phages J5a and F16Ba are about 50% longer than PlyG, the most known anthrax lysin (351 aa vs. 233 aa), and are only slightly similar to PlyG (27% coverage and 23% identity; Fig. 1a, b). Results of analysis by SignalP 5.0 indicated that both of them are exported from the cytoplasm by the SecA-dependent transport pathway (Sec/SPI). The 27 aa-long signal peptides (SP) at their N-termini, by prediction, are cleaved off by a signal peptidase I (SPase I). Because SP-containing endolysins do not require holins to get access to the bacterial cell membrane, they are toxic to cells under overproduction conditions, causing cell lysis. Thus, in this study, the new endolysins’ genes were cloned in a modified form devoid of the SP-encoding sequence. The proteins obtained as the products of modified genes were designated LysJ and LysF, from the names of their parental phages J5a and F16Ba, respectively. Genes encoding LysJ-(His × 6) and LysF-(His × 6) lysins were expressed in *E. coli* BL21 (DE3) and purified by nickel-affinity chromatography. Both proteins migrated in the SDS-PAGE gel consistently with their predicted sizes: 37.6 kDa (LysJ-(His × 6)) and 37.7 kDa (LysF-(His × 6)) (Fig. 1a, b), as confirmed by Western blotting with anti-His-tag antibodies (Fig. 1a, b). Additionally, both proteins exhibited lytic activity against the *B. anthracis* cell wall in a zymogram assay (Fig. 1c).

### Antimicrobial activity of LysJ and LysF in spot tests

**Fig. 2:**
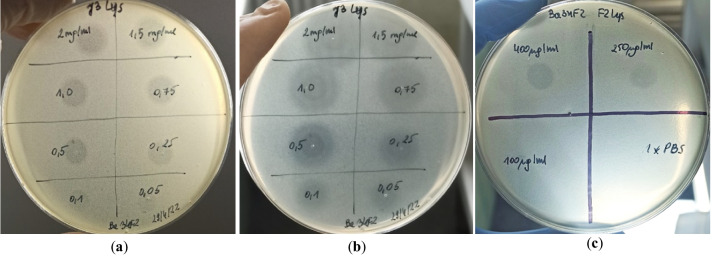
The lysis zones formed by LysJ (**a**, **b**) and LysF (**c**) on the lawn of *B. anthracis* 34F2 cells. The lysis was monitored after 6 (**a**) and 4 h (**c**) of plate incubation at 37 °C. Image (**b**) shows the LysJ-produced lysis zones after the prolonged incubation of the plate at 4 °C (72 h). Concentrations of the lysins in spots dropped on the bacterial lawns are indicated on plates in mg/ml (**a**, **b**) or µg/ml (**c**)

In the plate lysis assay, *B. anthracis* 34F2 strain used routinely to propagate phages J5a and F16Ba, turned out to be sensitive to both lysins (Fig. 2). However, the LysF concentration required for producing the visible lysis was higher than that of LysJ (Fig. 2c). In the case of LysJ, the diameters of lysis zones grew significantly with the time of plate incubation at 4 °C until about 72 h, indicating the diffusion of lysin and preservation of its activity under these conditions (Fig. 2b). LysJ was active in cell lysis of five virulent *B. anthracis* strains within the range of concentrations 0.5–2.0 mg/ml. In contrast to that, LysF was only slightly active in the lysis of cells of one of these strains (PZH) and only at a high concentration (0.4 mg/ml; images not shown). None of the tested lysins could form lysis zones on bacterial lawns of the other 33 *Bacillus* strains used in this study.

### Antimicrobial activity of LysJ and LysF in turbidity reduction assays

**Fig. 3:**
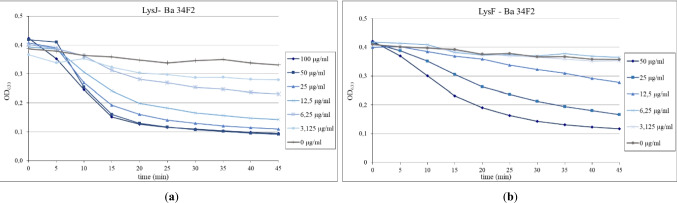
The influence of LysJ (**a**) and LysF (**b**) on the optical density of *B. anthracis* 34F2 cells suspension. The lysins at different concentrations were added to the bacterial cells suspended in 20 mM Tris–HCl, pH 8.0. The final concentrations of LysJ in wells were within the range of 3.125–100 µg/ml and of LysF 3.125–50 µg/ml. Bacterial suspensions with PBS instead of the proteins served as a negative control (0 µg/ml). All measurements were performed in triplicate

In the turbidity reduction assay of *B. anthracis* 34F2 cells suspension, a distinct concentration-dependent decrease of the optical density was observed for both lysins. LysJ at the concentrations 50 and 100 µg/ml caused a nearly 45% decrease in the suspension optical density (OD_630_) after 10 min of incubation. The minimal LysJ concentration that caused a noticeable reduction in OD_630_ was 3.125 µg/ml (Fig. 3a). In the case of LysF, the concentration of 50 µg/ml turned out to be the most effective, reducing OD_630_ by almost 50% after 15 min tests. However, the concentrations of 3.125 and 6.25 µg/ml were insufficient to cause any decrease in the cell suspension turbidity (Fig. 3b).

Testing the ability of LysJ and LysF to lyse the cells of each of the five virulent anthrax strains used in this study by the turbidity reduction assay showed the reduction of the optical density of cell suspensions in all cases tested. Under the tested conditions, both proteins significantly reduced the optical density of bacterial cell suspensions, from OD_630_ ~ 0.45–0.5 to ~ 0.15–0.2 on average (Supplementary Figures S1, S2). In the case of strains of *B. cereus*, *B. thuringiensis*, *B. mycoides*, *B.* sp*. Ba 813* + , and one strain of *B. subtilis*, very diverse, non-species-specific results were obtained, again, unlike in the spot test. The number of strains for which a decrease in OD_630_ could be detected (moderate or significant) was 6/10 of *B. cereus*, 9/10 of *B. thuringiensis*, 7/10 of *B.* sp. *Ba 813* + , 1/2 of *B. mycoides*, and one *B. subtilis.* Only some isolates remained insensitive. Selected graphs showing the best or moderate results of the OD_630_ reduction assay for the suspensions of cells of various *Bacillus* species strains are shown in Supplementary Figure S3.

The antibacterial activities of LysJ and LysF determined by the turbidity reduction assay and by the spot test differed, although LysJ displayed higher activity against different strains than LysF in all experiments performed, independent on the method used (see the summary in Table 2). In the spot tests, LysJ selectively killed only *B. anthracis* cells and was active against the vaccine strain as well as virulent strains. In that respect, its specificity resembled the specificity of its parental bacteriophage J5a. LysF could lyse in the spot test only the vaccine *B. anthracis* strain. Unexpectedly, in the turbidity reduction assay, both lysins appeared to be active not only against all *B. anthracis* strains tested but also against the majority of tested strains representing other *Bacillus cereus* group species, and also against one tested strain of *B. subtilis*, a species that does not belong to the *B. cereus* group (Table 2).

**Table 2:** The host range of phages J5a and F16Ba and the lytic spectrum of their modified endolysins, LysJ and LysF

Organism (number of strains)	Strain	ϕ J5a lysis	ϕ F16Ba lysis	Spot assay	Turbidity Reduction assay			References
				LysJ	LysF	LysJ	LysF	
*B. anthracis* (6)	Sterne 34F2^1^	** + **	** + **	** + **	** + **	** + + + **	** + + + **	(Dwyer et al. 2004; Negus et al. 2013)
	211^2^	** + **	** + **	** + **	-	** + + + **	** + + **	(Szymajda and Bartoszcze 2005; Negus et al. 2013)
	1153	** + **	** + **	** + **	-	** + + + **	** + + + **	(Cieślik et al. 2015; Graniak et al. 2020)
	1583^2^	** + **	** + **	** + **	-	** + + + **	** + + + **	(Szymajda and Bartoszcze 2005; Graniak et al. 2020)
	1584^2^	n.a	n.a	** + **	-	** + + + **	** + + **	(Szymajda and Bartoszcze 2005; Graniak et al. 2020)
	PZH^4^	** + **	** + **	** + **	+	** + + + **	** + + + **	
*B. cereus* (11)	ATCC 10872	-	-	-	-	** + **	** + **	
	ATCC 10876^3^	-	-	-	-	** + **	** + **	(Negus et al. 2013)
	ATCC 11778	-	-	n.a	n.a	n.a	n.a	
	ATCC 13472^3^	-	-	-	-	** + + **	** + + **	(Negus et al. 2013)
	ATCC 14579^ T 3^	-	-	-	-	** + **	** + **	(Negus et al. 2013)
	ATCC 19637^3^	-	-	-	-	-	-	(Negus et al. 2013)
	ATCC 23261^3^	-	-	-	-	** ± **	** ± **	(Negus et al. 2013)
	F16959^3^	-	-	-	-	-	-	(Dwyer et al. 2004)
	F17202^3^	-	-	-	-	** + + + **	** + + + **	(Dwyer et al. 2004)
	F17289^3^	-	-	-	-	** + + + **	** + + + **	(Dwyer et al. 2004)
	UW85^3^	-	-	-	-	-	-	(Dwyer et al. 2004)
*B. thuringiensis* (11)	ATCC 33679	-	-	-	-	** + + **	** + + **	(Negus et al. 2013)
	ATCC 35646^3^	-	-	-	-	** + + **	** + + **	(Dwyer et al. 2004)
	ATCC 10792	-	-	-	-	** ± **	** ± **	(Negus et al. 2013)
	ATCC 10792^ T^	-	-	-	-	** + + **	** + + **	(Dwyer et al. 2004)
	T07-019^3^	-	-	-	-	** + + **	** + + **	(Negus et al. 2013; Graniak et al. 2020)
	T07-128^3^	-	-	-	-	** + + **	** + + **	(Negus et al. 2013; Graniak et al. 2020)
	T07-146^3^	-	-	n.a	n.a	n.a	n.a	(Qi et al. 2001; Graniak et al. 2020)
	T07-151^3^	-	-	-	-	** + + **	** + + **	(Graniak et al. 2020)
	T07-155^3^	-	-	-	-	** + + **	** + + **	(Graniak et al. 2020)
	T07-202^3^	-	-	-	-	** + + + **	** + + + **	(Qi et al. 2001; Cieślik et al. 2015)
	#35^3^	-	-	-	-	** + + **	** + + **	(Cieślik et al. 2015)
*B. mycoides* (3)	ATCC 6462	-	-	-	-	-	-	
	ATCC 21929^3^	-	-	-	-	** + **	** + **	(Dwyer et al. 2004)
	K184^3^	-	-	n.a	n.a	n.a	n.a	(Czaban et al. 2004)
*B.* sp.* Ba 813* + (10)	#6 (I/2)^3^	-	-	-	-	** + **	** + **	(Negus et al. 2013; Graniak et al. 2020)
	#7 (II/3)^3^	-	-	-	-	** + **	** + **	(Graniak et al. 2020)
	#12 (S8553/2)^3^	-	-	-	-	** + + **	** + + **	(Dwyer et al. 2004; Graniak et al. 2020)
	#16 (PJ572)^3^	-	-	-	-	-	-	(Niemcewicz and Bartoszcze 2006a; Graniak et al. 2020)
	#17 (094)^3^	-	-	-	-	** + **	** + **	(Dwyer et al. 2004; Graniak et al. 2020)
	#21 (T1197-77)^3^	-	-	-	-	-	-	(Dwyer et al. 2004; Graniak et al. 2020)
	#28 (3)^3^	-	-	-	-	** + + + **	** + + + **	(Niemcewicz and Bartoszcze 2006a; Graniak et al. 2020)
	#30 (1B)^3^	-	-	-	-	** + + **	** + + **	(Graniak et al. 2020)
	#31^3^	-	-	-	-	** + **	** + **	(Graniak et al. 2020)
	#3403^3^	-	-	-	-	** + **	** + + **	(Dwyer et al. 2004)
*B. subtilis*	ATCC 6633^3^	-	-	-	-	** + + **	** + + **	(Negus et al. 2013)

In the columns with phage lytic activity and the results of spot tests the “ + ” or “- “ signs indicate the presence or lack of lysis zones, respectively. In the column with the results of the turbidity reduction assay, the “ + ” sign indicates the degree of the optical density reduction: slight ( +), moderate (+ +), or intense (+ + +)

*n.a.* non-applicable

Notes: ^1^Strain isolated from “Antraphyl” vaccine by Phylaxia-Sanofi (serial no. 021OE2)

^2^Strain acquired by the Military Institute of Hygiene and Epidemiology (MIHE) from the Institute of Veterinary Hygiene in Białystok, the field office in Łomża, Poland

^3^Strains acquired by MIHE from the Institute of Molecular Biology and Medicine (IMBM) at the University of Scranton, PA, USA

^4^Strain acquired by MIHE from the National Institute of Public Health – National Institute of Hygiene, Poland

### Analysis and comparison of LysJ and LysF amino acid sequences and 3D structures modeling

**Fig. 4:**
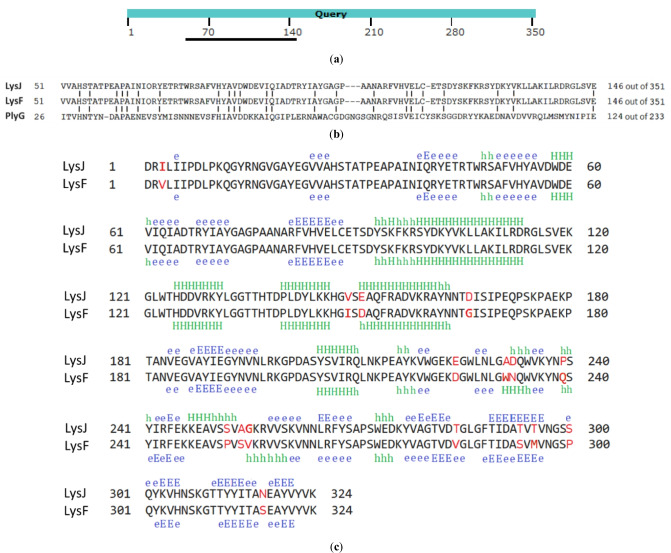
Schematic alignment of the amino acid sequences of J5a (QOQ37201.1) and F16Ba (QOQ37151.1) phage endolysins (Query, 351 aa) with phage Gamma PlyG lysin (WP_001982889.1) in BLASTp. The black line indicates the PlyG region with the highest similarity to the query sequences located within the catalytic domain region (EAD) (**a**). The alignment of amino acid sequences of the highest similarity regions of J5a and F16Ba endolysins and PlyG. Vertical bars indicate identical amino acid residues in all three lysins at the given position (**b**). Comparison of the amino acid sequences and predicted secondary structures of LysJ and LysF. The numbers on the sides indicate the range of amino acid residues. Differing amino acid residues are marked in red. Based on the HHpred results, the structure of the β-strand (E) was marked in blue, and the structure of the α-helix (H) in green. The empty regions in between include a coiled-coil structure formed by two or more mutually coiled α-helices. Uppercase and lowercase letters indicate high and low prediction certainty, respectively (Gabler et al. 2020) (**c**)

The LysJ and LysF were identified as N-acetylmuramoyl-L-alanine amidases sharing 95% of their amino acid sequences, like their parental endolysins (Fig. 4). Although their amino acid sequences are highly similar, some of the differing sequence fragments contain amino acid residues with significantly different properties (Fig. 4c), suggesting that some of these differences may account for the differences in the lytic activity or specificity of both proteins. For example, serine (S) residues at positions 252 and 300 in LysJ are replaced by proline (P) in the corresponding positions in LysF, and the proline residue at position 239 of LysJ in the corresponding position of LysF is replaced with a glutamine residue (Q). In turn, a small hydrophobic alanine residue (A) at position 231 in LysJ is replaced by the large aromatic amino acid residue, tryptophan (W), in the corresponding position of LysF. Most of the amino acid sequence differences are located in the C-terminal domains of LysJ and LysF, i.e., downstream of the 170th amino acid residue (Fig. 4c). Comparison of the predicted LysJ and LysF secondary structures with the use of HHpred showed that some differences in the amino acid sequences of the two proteins correlate with the local differences in their secondary structures (Fig. 4c).

Results of our previous studies revealed the modular architecture of parental LysJ and LysF endolysins, with a highly conserved enzymatic domain (EAD) and a C-terminal cell-binding domain (CBD) (Nakonieczna et al. 2022). The EADs of LysJ and LysF contain motifs characteristic of zinc-containing Amidase_2 domains (pfam 01510, aa 88–167, 1.11e − 08, in both proteins) and PGRP (peptidoglycan recognition protein) conserved domains superfamily (cd065883, aa 29–170, 8.34e − 23), which includes Zn-dependent N-Acetylmuramoyl-L-alanine amidases, that cleave the amide bond between N-acetylmuramoyl and l-amino acids in bacterial cell walls. The CBD domains of both proteins contain motifs characteristic of bacterial SH3 domain homologs (smart00287, aa 212–263, 7.04e − 03). The similar domain structure of both lysins is indicated by the results of comparisons of their predicted secondary structures with known structures of proteins from the PDB database. The closest structural homologs of LysJ and LysF EAD domains include amidase and peptidoglycan binding domains of bifunctional major autolysin AtlA of *Staphylococcus aureus* (4KNK_A, probability, 99.7%) and domains of a similar activity of, e.g., *Streptococcus pneumoniae* LytA autolysin (4IVV_A, probability, 99.4%), *Clostridium intestinale* N-acetylmuramoyl-L-alanine amidase involved in cell wall degradation (6SSC_A, probability, 99.3%). *Bacillus* or *Bacillus* phages lytic enzymes EADs of structural similarities to LysJ and LysF include the N-acetylmuramoyl-L-alanine amidase XlyA of *Bacillus subtilis* (3RDR_A, probability, 99.0%) and N-acetylmuramoyl-L-alanine amidase PlyL of *B. anthracis* prophage LambdaBa02 (1YB0_C, probability, 98.8%). The closest structural homolog of LysJ and LysF CBD domains is the CBD domain of *Listeria* phage A500 endolysin, which contains pseudo-symmetric SH3b-like repeats (6HX0_A, probability, 98.2%).

LysJ, LysF (sequences as shown in Fig. 4c), and PlyG (NCBI accession no. YP_338200) for comparison were further studied in the context of secondary and tertiary structure by in silico protein modeling. For this purpose, the amino acid sequences of three lysins were analyzed using SWISS-MODEL, a structure homology-modeling server, accessible via the Expasy web server (Guex 2009; Biasini et al. 2013; Bertoni et al. 2017; Bienert et al. 2017; Waterhouse et al. 2018; Studer et al. 2020). One, best-performing model for each lysin was used for the comparison.

**Fig. 5:**
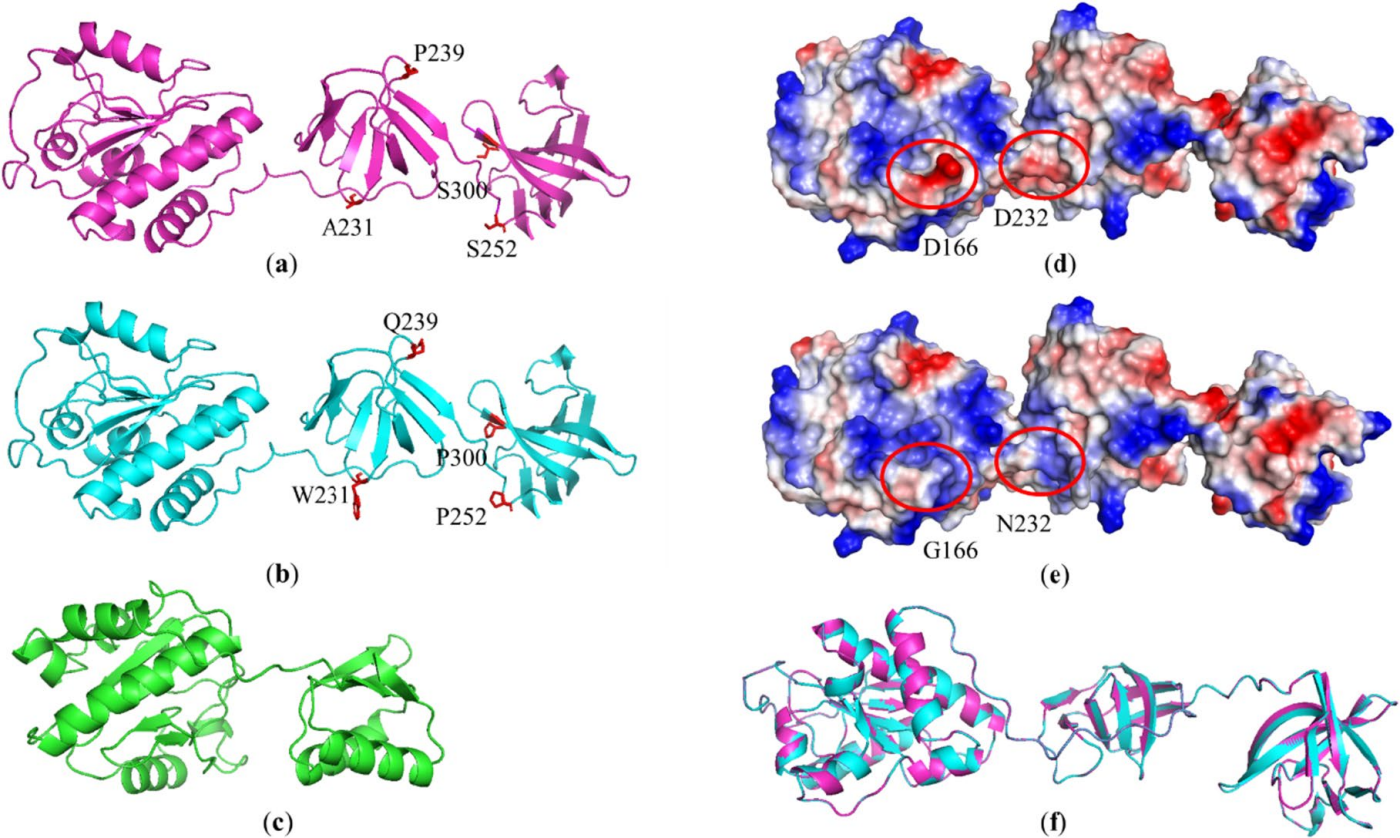
Comparison of the predicted protein structures of LysJ (**a**) and LysF (**b**) with phage Gamma PlyG lysin (**c**). On the view at the secondary and tertiary structures, the critical residues are highlighted as red sticks and labeled with the residue number for clarity. Comparisons of surface charges of LysJ and LysF are depicted in (**d**) and (**e**), respectively, where the red color indicates a negative charge and the blue color indicates a positive charge. Major charge differences are marked with red circles and labeled with the residue number. An overlay of the (**a**) and (**b**) structures is shown in (**f**)

The overall secondary and tertiary structures of LysJ and LysF lysins demonstrate a high similarity level, which stays in agreement with the previous analyses. Secondary structure composition is predominantly based on the anti-parallel β-sheets and coiled-coil, with only the N-terminal EAD domain containing α-helical elements surrounding centrally located pleated sheets of β-strands. Residues potentially responsible for the altered specificity of the lysins are located in a majority of the flexible regions, exposed to the protein surface (Fig. 5a, b). Comparison with the PlyG revealed essential differences in the length, spatial organization, and domain composition (Fig. 5c). In contrary to PlyG, which is composed of two domains, LysJ and LysF are composed of three distinct and globular domains joined with flexible linkers. The central and C-terminal structural domains build together one functional domain, i.e., the C-terminal CBD (as seen in Fig. 5a, b as the two smaller, β-strand-based parts of the proteins). The N-terminal EAD domain, however, shows high structural similarity in all three proteins (Fig. 5a, b, c; the left part of the proteins). Surface charges of LysJ and LysF demonstrate high similarity levels (Fig. 5d, e). However, subtle and singular negatively charged residues, such as D166 and D232, potentially form a platform for altered features, including specificity. The direct overlay of both structures (Fig. 5f) underlines the high similarity of the secondary and tertiary structures of LysJ and LysF.

## Discussion

In this study, we obtained, purified, and tested for lytic activity two new lysins, LysJ and LysF, which were acquired as derivatives of *B. anthracis* J5a and F16Ba phage endolysins depleted of the N-terminal signal peptides (Nakonieczna et al. 2022). We showed previously that the J5a and F16Ba phage endolysins are similar at the amino acid sequence level. Both of them contain a signal peptide at their N-termini, the enzymatic amidase domain (EAD), and the C-terminal cell wall binding domain (CBD). The sizes of LysJ and LysF lysins (about 37 kDa) and their domain composition conform with the typical sizes (25–40 kDa) and domain composition of lysins from phages that infect Gram-positive bacteria and are, in most cases, composed of one EAD and one CBD domain (Fischetti 2010). However, the amino acid sequences of both endolysins are remarkably different from those of other known anthrax lysins, amidases PlyG and PlyPH, and lysozyme PlyB. Additionally, LysJ and LysF differ in the combination of their domain activities from other analyzed anthrax lysins. The EAD domains of LysJ and LysF are similar to the lysin EAD domains of zinc-dependent amidase activity, which were also identified in PlyG and other *B. anthracis* targeting lysins (Schuch et al. 2002; Low et al. 2005; Mehta et al. 2013). However, the predicted secondary structures of LysJ and LysF CBDs adopt the arrangements different from those of other analyzed *B. anthracis* targeting N-Acetylmuramoyl-L-alanine amidases. They are highly similar to the structures of CBDs of *Listeria* phage A500 lysin, Ply500, and *Bacillus* phage PBC5 lysin (Nakonieczna et al. 2022), although similarities between the CBDs of LysJ and LysF and the CBDs of Ply500 and PBC5 at the amino acid sequence level are marginal. The CBD domains of Ply500 and PBC5 lysins comprise two copies of the beta-barrel SH3b-like repeats (Broendum et al. 2018; Lee et al. 2019; Shen et al. 2021). Meanwhile, the CBD domain of PlyG endolysin, from the Gamma phage, a *Wbetavirus* closely related to the J5a and F16Ba phages, is composed of a central antiparallel four-stranded β-sheet with a helix flanking each side and was assigned to the α/β multimer type (Broendum et al. 2018). The SH3b-like repeats containing CBD domains are commonly found in endolysins of Gram-positive bacteria-infecting phages (Nelson et al. 2012; Etobayeva et al. 2018), including *B. cereus-group* phage endolysins (Leprince et al. 2020; Nakonieczna et al. 2022), but the structural basis for cell wall recognition by them differ significantly from those of CBD domains of α/β multimer type (Lee et al. 2019; Shen et al. 2021; McGowan et al. 2012; Broendum et al. 2018). Thus, LysJ and LysF may potentially enrich the repertoire of lysins targeting *B. anthracis* cells with specificity to a new cell wall epitope.

Despite the high similarity of LysJ and LysF encoding sequences, both proteins differed in the lytic activity against the cells of *Bacillus* strains used in this research. It could be assigned to their slightly different 2D structures and differences in their amino acid sequences that are located mostly in the predicted CBD regions, suggesting distinct affinity of both lysins to *Bacillus* cell walls (Fig. 4c). However, after analyzing the predicted tertiary structures of these two proteins, it may be assumed that the differences in their specificity may be influenced not so much by the discrepancies in their predicted secondary structures, but by the difference in their surface charges (Figs. 4c and 5d, e). One may expect a correlation between an enzymatic activity of a protein and its charge, especially of EAD, and also the electrostatic nature along with the shape contribute to the functional protein interactions and catalytic specificities (Oliveira et al. 2013; Etobayeva et al. 2018).

Major differences in the enzymatic activity between LysJ and LysF were seen in the zymogram assay and in the spot tests on solid media with the cell layers of *B. anthracis* 34F2 and five virulent anthrax strains. LysJ showed a higher level and a broader spectrum of activity than LysF in these tests. Longer incubation times did not improve the results on the plates where the lysis zones were not observed, even though the plate method seems to become more sensitive with increasing incubation time (Etobayeva et al. 2018). More similar results for both lysins were obtained in the optical density (OD) reduction assay. Both enzymes significantly reduced the density of *B. anthracis* 34F2 strain suspensions even at very low concentrations, much lower than those used in the spot test (Fig. 3). However, the decreases in the optical density of the virulent *B. anthracis* cells suspensions occurred later than in the case of the vaccine isolate suspension. Perhaps the reason for this delayed action is the presence of a capsule in virulent strains, where poly-γ-D-glutamic acid residues may hinder the contact of lysins with the cell wall. The vaccine *B. anthracis* strain lacks the pXO2 plasmid encoding the genes of this capsule, making it sensitive to phagocytosis in the infected organism (Brey 2005).

Differences in the results of LysJ and LysF lytic activity and specificity testing with the use of spot test and cell density reduction assay suggest the existence of factors determining the different accessibility of peptidoglycan for lysins in cells grown under different culture conditions. Indeed, while *B. anthracis* cells for the spot test were derived from dense, overnight, stationary phase cultures grown in LB broth, the cells for the optical density reduction assay were derived from the exponentially grown cultures and suspended in 20 mM Tris–HCl. *B. anthracis* cells can have two structures on their surface, namely the S-layer (surface layer) and the capsule (Fouet and Mesnage 2002). The S-layer found in many bacteria species, including members of the family *Bacillaceae*, completely covers the cell above the peptidoglycan layer and comprises two major proteins, Sap and EA1, produced sequentially from chromosomal genes (Mignot et al. 2002). During the logarithmic phase of bacterial growth, Sap protein forms the S-layer, which is relatively ordered and flexible (Couture-Tosi et al. 2002). During the early stationary phase, protein networks formed by both proteins, Sap and EA1, begin to be present in the S-layer. The protein network built of the EA1 protein creates a finer lattice with cross-links oriented in different directions (Couture-Tosi et al. 2002). Conceivably, due to the aforementioned differences, the peptidoglycan of cells from older cultures is more difficult to reach for the lysins, which may be a reason for differences in our results obtained by different methods.

Although in the case of LysJ and LysF, the method of assessing the lytic activity by the optical density reduction assay was found to be more sensitive than the spot test, the results obtained for other lysins using both discussed assays do not always follow this pattern. For example, Etobayeva et al. (2018) noted the opposite situation in the case of *Bacillus* lysins PlyP56 and PlyN74 which did not lyse cells of anthrax strains Ames35, and UM23 in suspensions but formed lysis zones in a spot test with the cell layers of these strains. The negative results of the optical density reduction assay were interpreted as an indication of the relatively weak activity of PlyP56 and PlyN74. Thus, based on the ability to significantly reduce the optical density of cell suspensions of most *Bacillus* strains tested, including all anthrax strains, the lytic activity of LysJ and LysF can be regarded as strong. At their maximal concentrations used, LysJ (at 100 µg/ml) and LysF (at 50 µg/ml) caused about a 45% decrease in the optical density of *B. anthracis* 34F2 suspension after 10 min of incubation. For comparison, in a similar experiment, lysins PlyB221 and PlyP32 derived from other *Bacillus cereus* group-infecting phages caused, respectively, up to 78% and 60% decrease in the optical density of suspension of *B. cereus* ATCC 10987 strain, closely related to *B. anthracis* (Helgason et al. 2000; Leprince et al. 2020).

Similarly to J5a and F16Ba phages, LysJ and LysF lysins did not produce clear zones on the plates with the cell layers of the *B. cereus*, *B. thuringiensis*, *B. mycoides*, and *B. subtilis* strains (Table 2). This initially suggested that they have the same host ranges as the phages. However, in the optical density reduction assay, the lytic range of the lysins appeared to exceed that of their parental phages. About 2/3 of the 33 tested strains from the *Bacillus* genus species were sensitive to LysJ and LysF in this assay. Similar observations have been made in the case of other lysins, including those derived from phages infecting *Bacillus* strains (Schuch et al. 2019; Leprince et al. 2020). Lysins often have a broader host range than their parental phages (O’Flaherty et al. 2005). In many cases, the binding spectrum of the CBDs of lysins spans even the entire genus of bacteria, as has been shown, for example, in studies with the GFP (green fluorescent protein)-labeled SH3b domains of staphylococcal lysins, indicating the ability of these lysins to recognize a conserved ligand in *Staphylococcus* cells (Gu et al. 2011).

Lysins with amino acid sequences highly similar to LysJ and LysF (over 90% identity) and with a signal peptide, like their parental J5a and F16Ba phage endolysins, are encoded only by six *Wbetavirus* genus phages deposited in GenBank (accessed March 29, 2023). None of them have been studied so far. Meanwhile, our ability to purify LysJ and LysF in the form of soluble enzymes with significant lytic activity against *B. anthracis* cells makes them promising candidates for in vivo studies. Phage-derived antimicrobials possess undeniable therapeutic competence and are successfully used against most infections caused by Gram-positive bacteria (Abdelrahman et al. 2021). Also, they could constitute a valuable tool in controlling biowarfare bacteria (Fischetti 2008), e.g., *B. anthracis*. Anthrax can threaten especially people of certain professions, e.g., veterinarians, scientists, butchers, tanners, or wool sorters (Misgie et al. 2015). The disease even got a “Woolsorters disease” nickname many decades ago (Tibbits 1881). Anthrax cases contribute significantly to infectious disease case statistics worldwide, with an annual global incidence of 2000–20,000 cases in the twenty-first century, according to the WHO estimates (Simonsen and Chatterjee 2022). Due to the high morbidity and mortality of infected people, low infectious dose, relative ease of production, and difficult detection and decontamination, anthrax bacilli, especially their spores, are among the most serious threats as biological weapons. Moreover, like many other microbes, they can develop resistance to antibiotics, which makes the development of lysin-based antimicrobials against them justified. The therapeutic properties of *B. anthracis* phage lysins have already been demonstrated in animal models. Fischetti (2008) showed that the administration of PlyG to mice challenged with *B. cereus* strain closely related to *B. anthracis* (RSVF1) increased the survival rate of the animals. Lysins LysJ and LysF proved their effectiveness in liquid cultures of different *Bacillus* species, especially virulent *B. anthracis* strains, and could be used for further studies to optimize their potential in fighting anthrax infections, either as a sole treating strategy or as a supplement to antibiotic therapy. Additionally, their EAD and CBD domains may serve as substrates for domain engineering and modifications to create new lysins with a desired activity, like, for example, chimeras of lysins of different origins (Bhagwat et al. 2020).

LysJ outperformed LysF in the ability to kill *B. anthracis* cells and the cells of other *Bacillus* sp. strains. Thus, LysJ may be considered a suitable candidate for further studies on its potential in treating human or animal infections.

## Supplementary Information

Below is the link to the electronic supplementary material.

Supplementary file1 (PDF 859 KB)

## Data Availability

All data generated or analyzed in this study are included in the published manuscript or its supplementary information file.
