# Supplementary material for: New bacteriophage-derived lysins, LysJ and LysF, with the potential to control Bacillus anthracis
Source: Appl Microbiol Biotechnol. 2024 Jan 9;108(1):76. doi: 10.1007/s00253-023-12839-z (PMC10776502; doi:10.1007/s00253-023-12839-z)
Supplement: Supplementary file 1 — Supplementary file1 (PDF 859 KB) [file 253_2023_12839_MOESM1_ESM.pdf]

Supplementary Information

# New bacteriophage-derived lysins, LysJ and LysF, with the potential to control *Bacillus anthracis*


Aleksandra Nakonieczna[1*], Małgorzata Łobocka[2]

[1] Biological Threats Identification and Countermeasure Center, Military Institute of Hygiene and Epidemiology, Puławy, 24-100, Poland

[2] Institute of Biochemistry and Biophysics of the Polish Academy of Sciences, Warsaw, 02-106, Poland

[*]corresponding author: aleksandra.nakonieczna@wihe.pl


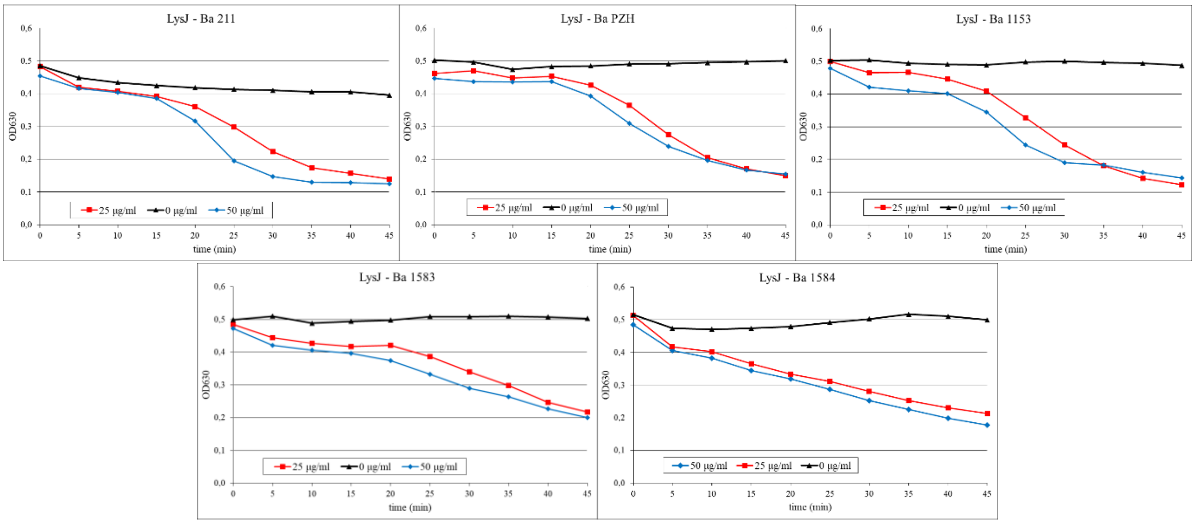


**Fig. S1** The effect of the lytic activity of LysJ in the optical density reduction study against virulent *B. anthracis* strains. LysJ at two concentrations, 25 and 50 µg/ml, was added to bacterial cells suspended in 20 mM Tris-HCl, pH 8.0. Bacterial suspensions with PBS instead of the proteins served as a negative control (0 µg/ml)

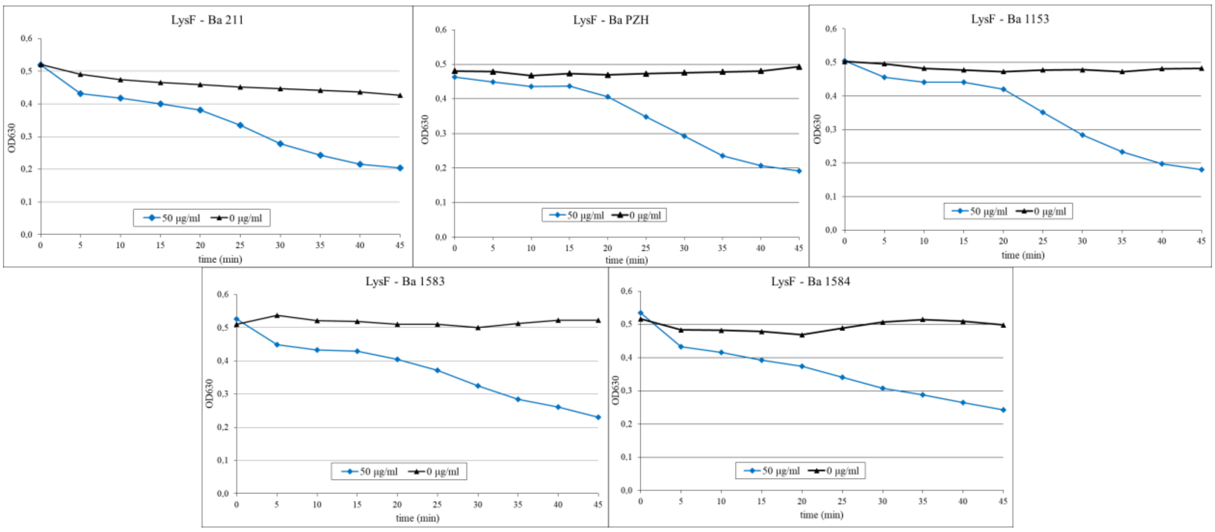


**Fig. S2** The effect of the lytic activity of LysF in the optical density reduction study against virulent *B. anthracis* strains. LysJ at a concentration of 50 µg/ml was added to bacterial cells suspended in 20 mM Tris-HCl, pH 8.0. Bacterial suspensions with PBS instead of the proteins served as a negative control (0 µg/ml)

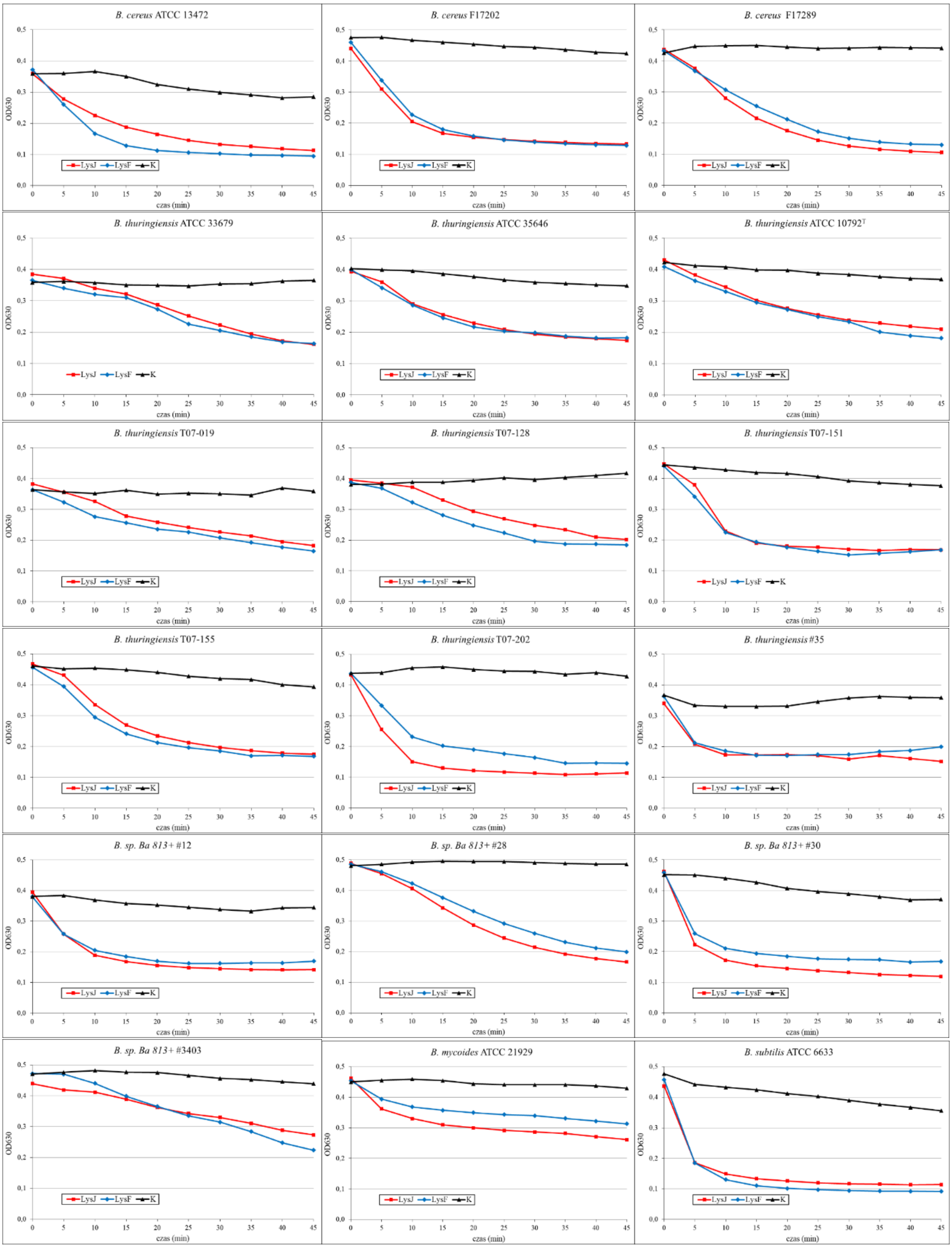


**Fig. S3** Selected graphs showing moderate and intense optical density reductions as a result of the lytic activity of LysJ and LysF against various *Bacillus* strains. The lysins were added individually to the bacterial cells suspended in 20 mM Tris-HCl, pH 8.0, to one final concentration of 50 µg/ml. Each graph shows the results obtained for both lysins. Bacterial suspensions with PBS instead of the proteins served as a negative control (0 µg/ml)
